# Ageing desexualizes the *Drosophila* brain transcriptome

**DOI:** 10.1098/rspb.2022.1115

**Published:** 2022-08-10

**Authors:** Antonino Malacrinò, Martin I. Brengdahl, Christopher M. Kimber, Avani Mital, Vinesh N. Shenoi, Claudio Mirabello, Urban Friberg

**Affiliations:** ^1^ Institute for Evolution and Biodiversity, Westfälische Wilhelms-Universität Münster, Münster, Germany; ^2^ Department of Agriculture, Università degli Studi Mediterranea di Reggio Calabria, Reggio Calabria, Italy; ^3^ IFM Biology, Linköping University, 581 83 Linköping, Sweden; ^4^ Department of Physics, Chemistry and Biology, National Bioinformatics Infrastructure Sweden, Science for Life Laboratory, Linköping University, 581 83 Linköping, Sweden

**Keywords:** ageing, brain, condition dependence, *Drosophila melanogaster*, senescence, sex-biased genes

## Abstract

General evolutionary theory predicts that individuals in low condition should invest less in sexual traits compared to individuals in high condition. Whether this positive association between condition and investment also holds between young (high condition) and senesced (low condition) individuals is however less clear, since elevated investment into reproduction may be beneficial when individuals approach the end of their life. To address how investment into sexual traits changes with age, we study genes with sex-biased expression in the brain, the tissue from which sexual behaviours are directed. Across two distinct populations of *Drosophila melanogaster,* we find that old brains display fewer sex-biased genes, and that expression of both male-biased and female-biased genes converges towards a sexually intermediate phenotype owing to changes in both sexes with age. We further find that sex-biased genes in general show heightened age-dependent expression in comparison to unbiased genes and that age-related changes in the sexual brain transcriptome are commonly larger in males than females. Our results hence show that ageing causes a desexualization of the fruit fly brain transcriptome and that this change mirrors the general prediction that low condition individuals should invest less in sexual phenotypes.

## 1. Introduction

Organisms commonly experience a decline in their physiological functioning with age [1]. This phenomenon, referred to as ageing or senescence, has a negative impact on the overall performance of organisms, reduces their ability to access and metabolize resources and results in elevated mortality and/or reduced reproductive rate [2–4]. Ageing, consequently, represents a degenerating somatic state that entails a shrinking pool of energy available for somatic maintenance and reproduction, and can hence be viewed as a decline in organismal condition [5].

Condition plays a central part in how life-history theory predicts organisms should allocate resources between current reproduction (through sexual traits) and somatic maintenance (investment in future reproduction) to maximize fitness [6,7]. In general, theory predicts that low-condition individuals should invest less than high-condition individuals into sexual traits, since condition determines an individual's ability to pay the viability costs associated with sexual trait exaggeration [8,9]. Empirical tests largely support this prediction (e.g. [10–14]).

When ageing reduces condition, the relative investment of low (old) and high (young) condition individuals in sexual traits becomes more difficult to predict, since the elevated risk of dying associated with ageing may select for increased investment at old age [15–17]. Predicting how investment will change with age is potentially also complicated by constraints, since (i) individuals may not be able to discriminate between a reduction in condition caused by inadequate access to resources and low condition owing to ageing, (ii) the weakening force of selection with age may prevent optimization of investment at old age, and (iii) the degenerate soma of old individuals may no longer fully respond to elevated investment.

To address how the reduction in condition that follows from ageing influences investment into sexual traits, we study changes in the sexual brain transcriptome (SBT, i.e. genes with sex-biased expression in the brain) between young and old *Drosophila melanogaster* males and females. The size of the SBT should provide an unbiased estimate of the current investment level into sexual traits (sexual and reproductive behaviours), since sex-biased gene expression is the first step by which energy is channelled towards sexual traits, gene expression can rapidly change in response to the environment, and because a degenerate soma (owing to age) should not limit its expression. Our results show a distinct reduction in the SBT with age owing to desexualization of gene expression in both sexes, and hence support the prediction from general condition dependence theory.

## 2. Results

We compare the SBT of young (5 days) and moderately old (25 days) flies in two populations of *D. melanogaster* (LH_M_ and Dahomey). On adult day 5 flies are fully sexually mature [18] and by adult day 25 they have typically clearly senesced [19,20]. Further, on adult day 25, most flies are still alive, limiting the opportunity for selective deaths to influence the mean phenotype of old individuals [21,22]. We verified that ageing (in terms of reproductive output) occurs by this age in the populations we study (electronic supplementary material, file S1, figure S1), and that mortality until adult day 25 is relatively low in both populations (electronic supplementary material, file S2, figure S2).

### (a) Sex-biased gene expression declines with age

The SBT evolves rapidly in *Drosophila* [23] and frequently shows substantial differences between *D. melanogaster* strains ([24] and references therein). Accordingly, we find that LH_M_ and Dahomey, which are differentiated with respect to both origin and culturing protocol (see Methods), share relatively few sex-biased genes (electronic supplementary material, file S2, figure S3). This difference between the populations may, at least partly, also result from that samples from the Dahomey population were more variable than those from the LH_M_ population, giving us less statistical power to identify sex-biased genes in Dahomey (electronic supplementary material, file S2, figure S4). The populations also differ with respect to how sex and age influence genetic variation, as shown by how these two factors associate to the 10 first principal components from a principal components analysis (electronic supplementary material, file S2, figures S5–S7). To test for changes in the SBT with age, we first compare the number of male-biased (MB) and female-biased (FB) genes between young and old flies in each population separately. Independently of adjusted *p*-value cut-off, we find significantly fewer MB and FB genes in old compared to young flies in both populations (except for Dahomey MB genes at adjusted *p*-value cut-off of 0.001, which left few significant MB genes at both ages), suggesting a general desexualization of the SBT with age (electronic supplementary material, file S2, table S1a–S1c). These results corroborate earlier findings, where the number of sex-biased genes, in general, have declined when condition has been experimentally reduced either through resource limitation [25–28] or mutational load [29]. When comparing the coefficient of variation for gene expression between young and old flies, we find that it is lower in old LH_M_ females, while it is higher in old LH_M_ males and in both sexes of old Dahomey flies (electronic supplementary material, file S2, figure S4). The reduced detected number of sex-biased genes in old flies could thus, in Dahomey, but presumably not in LH_M_, at least partly result from elevated variance in gene expression at old age.

**Figure 1.**
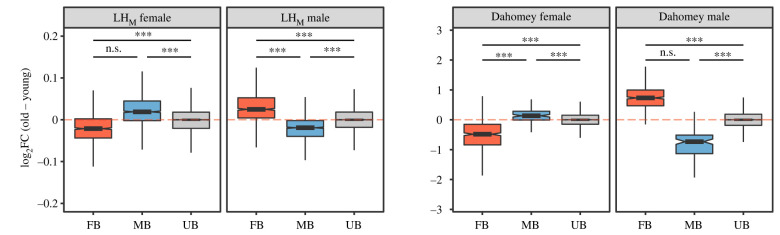
Differential gene expression between old and young flies in LH_M_ and Dahomey, with genes grouped on the *x*-axis according to their sex-bias in young flies (FB = female biased, MB = male biased, UB = unbiased), in each sex separately. Values on the *y*-axis represent log_2_ fold changes with age. Pairs of boxes are labelled with asterisks whenever their absolute distances from the 0-axis differ significantly. This was tested with a Bayesian linear model (***pMCMC < 0.001; **pMCMC < 0.01; *pMCMC < 0.05; n.s. pMCMC > 0.05). Note that graphs are on different scales. (Online version in colour.)

To study how the SBT changes with age within each sex separately, we next analyse how the expression level of genes identified as MB and FB in young flies changes with age in each sex. In males from both populations, expression of MB genes on average declines with age in comparison to unbiased (UB) genes, while expression of FB genes on average increases with age compared to UB genes (electronic supplementary material, file S2 table S2a–S2b). In females, we find the opposite pattern in both populations. Here, the expression of FB genes on average declines with age in comparison to UB genes, while expression of MB genes on average increases with age compared to UB genes (electronic supplementary material, file S2 table S2a–S2b). We note that these results are not influenced by changes in expression variation in genes with age, since such expression changes are adjusted for in our analyses (see Methods for details). These results thus show that desexualization of gene expression occurs in both sexes of both populations with respect to MB as well as FB genes (figure 1), and thus confirms that there are fewer sex-biased genes in old brains. Somewhat unexpectedly, but largely in line with Malacrinò *et al*. [29], we find that desexualization of FB genes is larger than that of MB genes in LH_M_ males and in Dahomey females. There is no difference in response between FB and MB genes in LH_M_ females and in Dahomey males (figure 1; electronic supplementary material, file S2, table S2a–S2b). We find similar results if we include the genes that are significantly sex-biased only at old age (electronic supplementary material file S2, table S2c–S2d), and if we take the tissue specificity of genes into account (electronic supplementary material, file S2, table S2e–S2f). Collectively, these results consistently indicate that ageing causes reduced, rather than increased, investment into the SBT.

### (b) Desexualization with age depends on the degree of sex-biased expression

**Figure 2.**
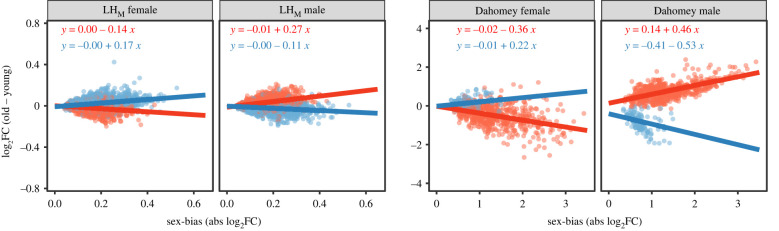
Changes in expression with age for female-biased (FB) and male-biased (MB) genes in each sex in relation to the degree of sex-bias. The *x*-axis shows the degree of sex-bias for MB and FB genes and the *y*-axis their log_2_ fold changes in expression with age. Note that graphs are on different scales. (Online version in colour.)

Traits experiencing strong sexual selection are expected to evolve exaggerated sexual dimorphism and therefore elevated condition-dependent expression [11,12]. We test this prediction and find that the desexualization (i.e. reduced expression of FB MB genes and elevated expression of MB FB genes in females males) of gene expression with age increases with increasing level of sex-bias, a pattern that holds for both FB and MB genes, in both sexes and populations (all eight *p* ≤ 0.02; electronic supplementary material, file S2, table S3a–S3b; figure 2). These findings align with earlier results, where condition was manipulated either through access to resources [25] or genetic quality [29]. In LH_M_ males, the association between the degree of sex-bias and desexualization with age was stronger for FB than MB genes (*p* < 0.0001; electronic supplementary material, file S2, table S3a). There was no difference in association with degree of sex-bias between FB or MB genes in LH_M_ females, or in either sex in Dahomey (all *p* > 0.21; electronic supplementary material file S2, table S3a–S3b).

### (c) Sex-biased genes show heightened age-dependent expression

**Figure 3.**
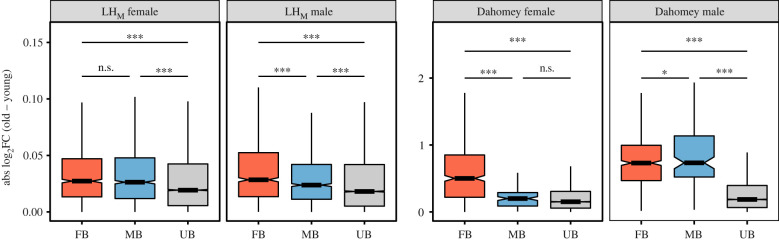
Absolute change in expression with age. Absolute log2 fold changes in gene expression between old and young flies in LH_M_ and Dahomey (*y*-axis), with genes grouped according to their sex-bias in young flies (*x*-axis) in each sex separately. FB = female biased, MB = male biased, UB = unbiased. Tests for differences between groups were conducted using a Bayesian linear model (***pMCMC < 0.001; **pMCMC < 0.01; *pMCMC < 0.05; n.s. pMCMC > 0.05). Note that graphs are on different scales. (Online version in colour.)

The theory of condition-dependent expression of sexual traits predicts they should evolve heightened sensitivity to changes in condition [6,7]. To test if the SBT shows heightened age-dependent expression, we compared the change in absolute expression of FB and MB genes to UB genes. We used absolute change since there is no prediction with respect to direction of expression changes for individual UB genes with age, and since above analyses show that their expression increases as often as it decreases with age (figure 1; electronic supplementary material file S2, table S2a–S4f). We find that both MB and FB genes change significantly more than UB genes with age in both sexes and populations, except for MB genes in Dahomey females (figure 3; electronic supplementary material, file S2, table S4a–S4b). These results thus show that sex-biased genes in general are more sensitive than UB genes when condition is reduced through ageing. In accordance with above analyses, we also find that the absolute change of FB genes is larger than that of MB genes in LH_M_ males and in Dahomey females, and that there is no difference in change between FB and MB genes in LH_M_ females. In contrast to the above results, there is a larger absolute change in MB than FB genes in Dahomey males (figure 3; electronic supplementary material, file S2, table S4a–S4b).

### (d) Sex-biased genes are in general more sensitive to ageing in males

Since sexual selection in general acts stronger on males than females [30,31], sexual traits are expected to evolve higher condition dependent expression in males. We use three approaches to test this prediction: (i) by taking direction of change into account with UB genes as reference; (ii) by comparing absolute change; and (iii) by comparing the slopes by which desexualization of expression occurs with increasing sex-bias. In Dahomey, we find that both MB and FB genes are more sensitive in males than MB and FB genes in females (in all four possible comparisons), both when taking direction into account and when analysing absolute change (all pMCMC < 0.001; figures 1 and 3; electronic supplementary material, file S2, tables S2b, S4b). In Dahomey, we also find that desexualization with age increases faster with increasing level of sex-bias for FB genes in males compared to both FB and MB genes in females (both *p* < 0.05), while there is no significant difference in slope between MB genes in males and either MB or FB genes in females (both *p* > 0.10; figure 2; electronic supplementary material, file S2, table S3b). Taking direction into account, in LH_M_ we find that expression of FB genes in males is more sensitive to age than both FB and MB genes in females (both pMCMC < 0.001), while there is no difference between MB genes in males and FB or MB genes in females (both pMCMC > 0.29; figure 1; electronic supplementary material, file S2, table S2a). When focusing on absolute change for LH_M_, we again find that FB genes in males are more sensitive than both FB and MB genes in females (both pMCMC = 0.003), while MB genes in males are less sensitive to age than both FB and MB genes in females (both pMCMC ≤ 0.007; figure 3; electronic supplementary material, table S4a). We further find that desexualization with age increases faster with increasing sex-bias in males for FB genes than both MB and FB genes in females (both *p* < 0.0001), while there is no difference between MB genes in males and FB genes in females (*p* = 0.09), and MB genes show increased desexualization in females compared to males (*p* = 0.0003; figure 2; electronic supplementary material, file S2, table S3a). While not true for all comparisons, these results largely support that the size of the SBT is more sensitive to ageing in males than in females.

### (e) Ageing and expression changes in the sex determination and nutrient sensing pathways

Condition-dependent sex-specific changes of gene expression are presumably regulated by an interaction between genes in the sex-determination pathway and genes in the nutrient sensing (insulin/insulin-like signalling and target of rapamycin (IIS/TOR)) pathway [32–34]. We therefore tested if genes in these pathways, as well as a subset of genes acting downstream of the IIS/TOR pathway (following [32]), change their expression with age. Since *double-sex* (*dsx*), a transcription factor at the tip of the sex determination pathway with sex-specific isoforms that regulate much of the sexual transcriptome, has the capacity to change gene expression in the opposite direction in the sexes [24,35–37], we hypothesized this gene could play an important part in generating the patterns we observe. Point estimates support this scenario, since males and females from both populations have lower expression of this gene when old, but in no case was the difference significant (electronic supplementary material, file S3). Along these lines we find relatively few significant changes with age across sexes and populations in the set of candidate genes we test. Significant changes were primarily limited to the ISS/TOR pathway and downstream genes in Dahomey males, a result in line with the generally larger change in expression of sex-biased genes with age in this sex and population (figure 1; electronic supplementary material, file S3). This is also consistent with the fact that direct manipulation of this pathway has a larger effect on the male than the female transcriptome [32].

## 3. Discussion

Our analyses of the *Drosophila* SBT in young and moderately old flies show that (i) old brains have fewer sex-biased genes than young brains, (ii) in both sexes expression of male- and female-biased genes show desexualization as they converges toward an intermediate sexual phenotype with age, (iii) sex-biased genes in general show heightened age-dependent expression that scales with the degree of sex-bias, and (iv) sex-biased genes are commonly more sensitive to ageing in males than females. These results align closely to predictions from the condition dependence theory of sexual trait expression [6,7,38–41] and to results obtained when condition of flies and beetles has been experimentally reduced through manipulation of resource availability [25,26] or genetic quality [29,42]. Our study hence supports the idea that ageing reduces investment into sexual phenotypes in a similar manner to reduced condition. At the same time, our results are incompatible with the idea that flies increase their investment into sexual phenotypes when they grow old, although a caveat with this conclusion is that terminal investment may be limited to the very last period before death, an age that varies between individuals and was not investigated here.

The populations we study differ with respect to both their ancestry and how they, for many hundreds of generations, have been selected to perform according to their different laboratory culturing protocols (see Methods for details). Since sex-biased genes evolve rapidly in *Drosophila* [23], it is thus not surprising that the populations differ with respect to the genes we identify as sex-biased. The fewer sex-biased genes identified in the Dahomey population, as well as their higher sex-bias, presumably also results from the Dahomey samples showing higher variability in gene expression, and thus, are associated with lower statistical power. The higher variability of the Dahomey data is potentially explained by this population hosting more genetic variation (its culturing protocol is less controlled), but it may also result from these samples showing lower complexity in terms of unique sequence reads. The fact that we, across populations and statistical power, find the same general patterns with respect to how expression in sex-biased genes change with age is nevertheless reassuring.

Our study provides no answer to what molecular mechanism downregulates the SBT with age since few candidate genes in the sex determination and the nutrient sensing pathways changed expression significantly with age and no consistent patterns across populations were found. A possible explanation for this result is that the regulatory signal supressing the SBT is smaller in magnitude than we had power to detect here. This interpretation aligns with the fact that most sex-biased genes individually show a rather small change in expression with age, although they show a consistent pattern as a group. An alternative possibility is that sexualization of the SBT is regulated through titres of juvenile hormone (JH). In *Drosophila* females, JH regulates allocation into reproductive activities and influences lifespan [43], and in males, it influences courtship activity [44]. In stag beetles, JH has further been shown to interact with *dsx* to produce sexually dimorphic structures during development [45]. It is, therefore, possible that JH modulates the effect of *dsx*, and that this molecular mechanism regulates the size of the SBT. Because JH is produced in the corpora allata, a gland located outside the brain, our current data does not allow testing for expression changes of genes involved in the biochemical pathway that produces JH.

The general desexualization of the SBT with age we document in *Drosophila* presumably extends to other species and somatic tissues [46]. In mice, it has been observed that the number of sex-biased genes in the hippocampus is lower in old individuals compared to young adults [47], and in humans, genes involved in energy metabolism have a feminized expression pattern in old males across brain regions [48]. In terms of other tissues, studies show that the mouse liver transcriptome is feminized in old males [49,50] and that this also is the case after a short-term restriction of calorie intake [51], supporting the idea that ageing and reduced general condition regulate sex-biased gene expression through a common mechanism.

The weakening force of selection with age is expected to result in idiosyncratic changes in gene expression with age in different species, but a few general patterns have been observed across taxa [52]. Future analyses on sexualized tissues in other species will answer if desexualization belongs to the short list of gene expression hallmarks of cellular ageing.

## 4. Methods

### (a) Fly populations

In our experiments, we used two laboratory-adapted populations of *D. melanogaster* (Dahomey and LH_M_) that differ with respect to both their origin and laboratory culturing protocol. Dahomey originates from flies collected in Benin (Africa) over 50 years ago. Ever since, it has been kept in cages as a large, outbred population, with overlapping generations and in constant conditions (at 25°C on a 12 L ∶12 D cycle, 60% relative humidity, and on a standard yeast-sugar medium) [53]. Flasks with food are cycled into the cages every week and cycled out after four weeks. Larval densities are not controlled, and flies can contribute with offspring until their natural death. LH_M_ originates from California (United States) and has for the last approximately 700 generations been cultured at a large, fixed population size (1792 breeding adults) on a strict 14 day discrete generation cycle and in constant conditions (at 25°C on a 12 L∶12 D cycle and standard cornmeal-molasses-yeast medium, and at 60% relative humidity the last approximately 200 generations) [54]. At the start of each generation, 56 vials are adjusted to each contain 150–200 eggs. In these vials, larvae compete over food, pupate and spend their early adult stages. On day 11, a randomized sample of 1792 adults from these vials are transferred in groups of 16 pairs into 56 fresh vials with a prescribed amount of live yeast on top of the culture medium. In these vials, males compete for fertilization opportunities and females compete for a limited supply of live yeast. On day 13, the adult flies are transferred into fresh vials where eggs are laid over an 18 hour period, completing the 14 day lifecycle. As a result of these differences in ancestry and laboratory selection regimes, we expect the LH_M_ and Dahomey populations to differ substantially, both with respect to reproductive schedules and how sexual selection has formed them.

### (b) Experimental procedures

To reduce phenotypic variation induced by larval condition and maternal effects, flies from the Dahomey population were taken out from the cages and reared in vials (20 vials with 16 pairs, eggs adjusted to 180 per vial) for several generations before the experiments were started. A similar procedure was not needed for LH_M_, since larval density is controlled every generation in this population. Experimental flies from each population were generated four months apart.

To produce experimental flies, 20 vials with 180 eggs were set up per population (on their respective food source). Ten days later we collected 80 vials with 15 adult males and females, separately for each population. After two days the flies were transferred into fresh vials. After two more days we collected the samples of young flies by flash-freezing flies from 15 randomly selected vials per population in liquid nitrogen. Because most flies emerge after nine days as juveniles, this sampling corresponded to adult day 5 for most flies. The remaining 65 vials with Dahomey flies were randomly grouped into five sets of 13 vials, and flies from each set were then placed in one of five plastic cages (390 flies per cage). LH_M_ flies remained in vials (density of 30 flies per vial). Every other day, cups with food were replaced in the cages with Dahomey flies, and LH_M_ flies were transferred into fresh vials. We scored for dead flies every time food was replaced. Twenty-four days after emergence, 45 pairs of Dahomey flies were aspirated out from each cage and sorted into three vials with 15 pairs each under light CO_2_ anaesthesia. The following day (25 after emergence), we collected the samples of old flies. The three vials from each of the Dahomey cages, and in total 15 vials with LH_M_ flies, were flash-frozen in liquid nitrogen. Any dead flies were removed without anaesthesia before freezing and freezing was conducted at the same time (14.00) for both young and old samples. All samples were stored at −80°C after collection. Brains were dissected under a stereomicroscope in RNAlater and stored at −80°C for less than 24 h before RNA extraction. In total we collected five independent biological replicates per population, sex and age (40 in total). Each replicate was made by pooling brains from 20 flies.

### (c) Library preparation and sequencing

Total RNA was extracted using an Agencourt RNAdvance Tissue kit (Beckman Coulter, US) following the producer's instructions. RNA samples were quantified using a Nanodrop 2000c spectrophotometer (Thermo Scientific, USA) and RNA quality and integrity were checked on a Bioanalyzer 2100 (Agilent, USA) using an RNA 6000 Nano kit. Immediately after quality control, RNA samples were used for library preparation using SENSE mRNA-Seq Library Perp Kit V2 (Lexogen, Austria), and dual-indexed with the i5 Dual Indexing Add-on Kit (Lexogen, Austria). Libraries were quantified using a Qubit 3.0 fluorometer (Thermo Scientific, USA), quality checked on a Bioanalyzer 2100 (Agilent, USA) using a DNA High Sensitivity kit, and pooled in equimolar ratios. The final pool was then sequenced on a single lane of an Illumina Novaseq 6000 S2 flow cell (50PE).

### (d) Bioinformatics and statistical analysis

Raw data was quality filtered and adaptors were trimmed using Trim Galore (v. 0.6.6 https://github.com/FelixKrueger/TrimGalore). Less than 0.4% of reads were lost on average during this step. Reads were mapped to the *D. melanogaster* reference genome (BDGP6) using STAR aligner (v. 2.6.1, [55]) and gene counts were extracted using featureCounts (v. 1.6.2, [56]). Details about parameters used throughout data pre-processing are available on GitHub (see: ‘Data and code availability’). All subsequent analyses were performed in the R statistical environment (v. 4.0.5) [57]. We used the *DESeq2* package (v. 1.30.0) [58] to normalize the raw counts, fit a model (separately for each population) using the formula *group* where the metafactor *group* includes all combinations of the two factors *Sex* (*Male, Female*) and *Age* (*A1* in young flies, *A2* in old flies), and perform subsequent contrasts. So, when contrasting by sex for young flies (age: *A1*), the contrast will be *FemaleA1* versus *MaleA1.* To contrast by age for a given sex (e.g. female), the contrast will be *FemaleA2* versus *FemaleA1*. In order to get more accurate log_2_ fold change (log2FC) values, we enabled DESeq2's default shrinkage method (by setting the parameter *betaPrior = TRUE*), which puts a zero-mean normal prior on the non-intercept coefficients in the model.

#### (i) Classification of sex-biased genes and test for changes in their numbers with age

We classified genes as sex-biased in young flies if they were differentially expressed between males and females (*p* < 0.05, false discovery rate corrected), and as FB or MB depending on if they were expressed more in brains dissected from female or male flies. Genes showing an absolute log2FC between males and females greater than or equal to 3.5 were removed in order to exclude genes with sex-limited expression [25,29]. Only two genes in the Dahomey population were affected by this filter, none in the LH_M_ population. All other genes were classified as UB, excluding from the analyses those that were not detected (zero counts) in any sample. We then repeated this classification separately for old flies to quantify the number of sex-biased genes for this group. These classifications were done separately for each population since sex-biased genes in brain tissue evolves rapidly in *Drosophila* [23] and often differ substantially between strains [24]. The number of sex-biased genes (FB and MB) in old flies was compared to the respective class in young flies using a *χ*^2^ test. The *p*-values from the *χ*^2^ tests were adjusted for multiple testing with Bonferroni correction.

#### (ii) Bayesian tests for changes in gene expression with age

To test whether sex-biased genes change their expression in relation to age, we first performed the contrast *Old*/*Young* with DESeq2 for each population and sex and obtained the corresponding log2FC values. In order to assess whether distributions of log2FC values change with respect to both sex and bias classification (MB, FB, UB), we then fitted a linear model using Bayesian Hamiltonian Markov chain Monte Carlo (MCMC) in the package *rstanarm* [59] using the formula *log2FC(Old/Young)* ∼ *group,* where *group* is a metafactor containing all unique combination of sex and bias class. We used the default weakly informative normally distributed priors for both the intercept (mean = 0, scale = 2.5) and coefficients (mean = 0, scale = 2.5), with autoscaling turned on, running four chains with 2000 iterations each and discarding the first 1000 as warm-up, for a total of 4000 observations. Comparisons between classes of genes were performed using the function *hypothesis* of the *brms* package [60], the equivalent of a two-tailed *p*-value (pMCMC) was estimated by calculating and doubling the frequency at which any of the 4000 observations was in disagreement with (had opposite sign to) the posterior estimate.

#### (iii) Accounting for tissue specificity when analysing changes in gene expression with age

In order to correct our results for tissue specificity, we fetched from FlyAtlas2, fragments per kilobase million (FPKM) values for each gene in different tissues in male and female flies. These values were sourced from 7-day-old adult flies (www.flyatlas2.org; [61]). We then used the FPKMs to calculate each gene's tissue specificity index *τ* [62]:

$$\tau =\frac{\sum_{i=1}^{N}(1-x_{i})}{N-1},$$

where *N* is the number of tissues in which the gene is expressed and *x_i_* is the FPKM value for the *i*th tissue normalized by its highest FPKM in any tissue. For each population, two linear models were then fitted separately for males and females with formula *log2FC(Old/Young)* ∼ *τ* and the residuals of the two models were used to perform the same Bayesian tests as above.

#### (iv) Tests for associations between degree of sex-bias and expression changes with age

We tested for a relationship between a gene's degree of sex-bias and its expression sensitivity to age by fitting a linear model with the *lm* function separately for FB and MB genes in each sex with the formula *log2FC(Old/Young)* ∼ *|log2FC(Young Male/Young Female)|*. The *p*-value associated with the regression coefficient was used to determine if this relationship is statistically significant. In order to test whether FB or MB genes decline at different rates in a given sex and population, we fit a separate linear model to the union of FB and MB genes using the formula *log2FC(Old/Young)* ∼ Bias*|log2FC(Young Male/Young Female)|. Similarly, in order to test whether genes decline at different rates in females or males for a given bias class (FB/MB) and population, we fit a linear model to all FB genes (either in males or females), and one to all MB genes, using the formula *log2FC(Old/Young)* ∼ Sex*|log2FC(Young Male/Young Female)|. In each of these last two tests, the *p*-values for the corresponding interaction terms were used to determine if two slopes (either MB/FB or Female/Male) are significantly different.

#### (v) Test for age-related changes in expression in the sex determination and the IIS/TOR pathways

When testing the effects of ageing on certain pathways, we redo the contrasts to calculate *log2FC(Old/Young)* as described above on a model with the formula ∼ Age separately by sex. In this case, the *p*-values are FDR-corrected only based on the number of tests performed in each given pathway.

## Data Availability

Raw sequencing data are available at NCBI SRA under the BioProject PRJNA759759. R code for all analyses and conda environment with package requirements are available on GitHub (See https://github.com/urbfr20/aging_brain/). Data is available in the electronic supplementary material [63].
